# Correction: Insights of the dental calculi microbiome of pre-Columbian inhabitants from Puerto Rico

**DOI:** 10.7717/peerj.3277/correction-1

**Published:** 2017-06-06

**Authors:** Tasha M. Santiago-Rodriguez, Yvonne Narganes-Storde, Luis Chanlatte-Baik, Gary A. Toranzos, Raul J. Cano

**Affiliations:** 1 Center for Applications in Biotechnology, California Polytechnic State University—San Luis Obispo, San Luis Obispo, CA, United States of America; 2 Biology Deparment, California Polytechnic State University—San Luis Obispo, San Luis Obispo, CA, United States of America; 3 Institute for Life Science Entrepreneurship, ATCC-Center for Translational Microbiology, Union, NJ, United States of America; 4 Center for Archaeological Investigations, University of Puerto Rico, San Juan, Puerto Rico; 5 Biology Department, University of Puerto Rico, San Juan, Puerto Rico

Christina Warinner was named in the Acknowledgements, which stated that she gave advice with regard to the methodology used in this work. Because the inclusion of her name in the Acknowledgements was done without her knowledge and because she did not concur with the methodology used in this paper, her name was removed from the Acknowledgements at her own request.

