# Supplementary figures and images for: Insights of the dental calculi microbiome of pre-Columbian inhabitants from Puerto Rico
Source: PeerJ. 2017 May 2;5:e3277. doi: 10.7717/peerj.3277 (PMC5417066; doi:10.7717/peerj.3277)

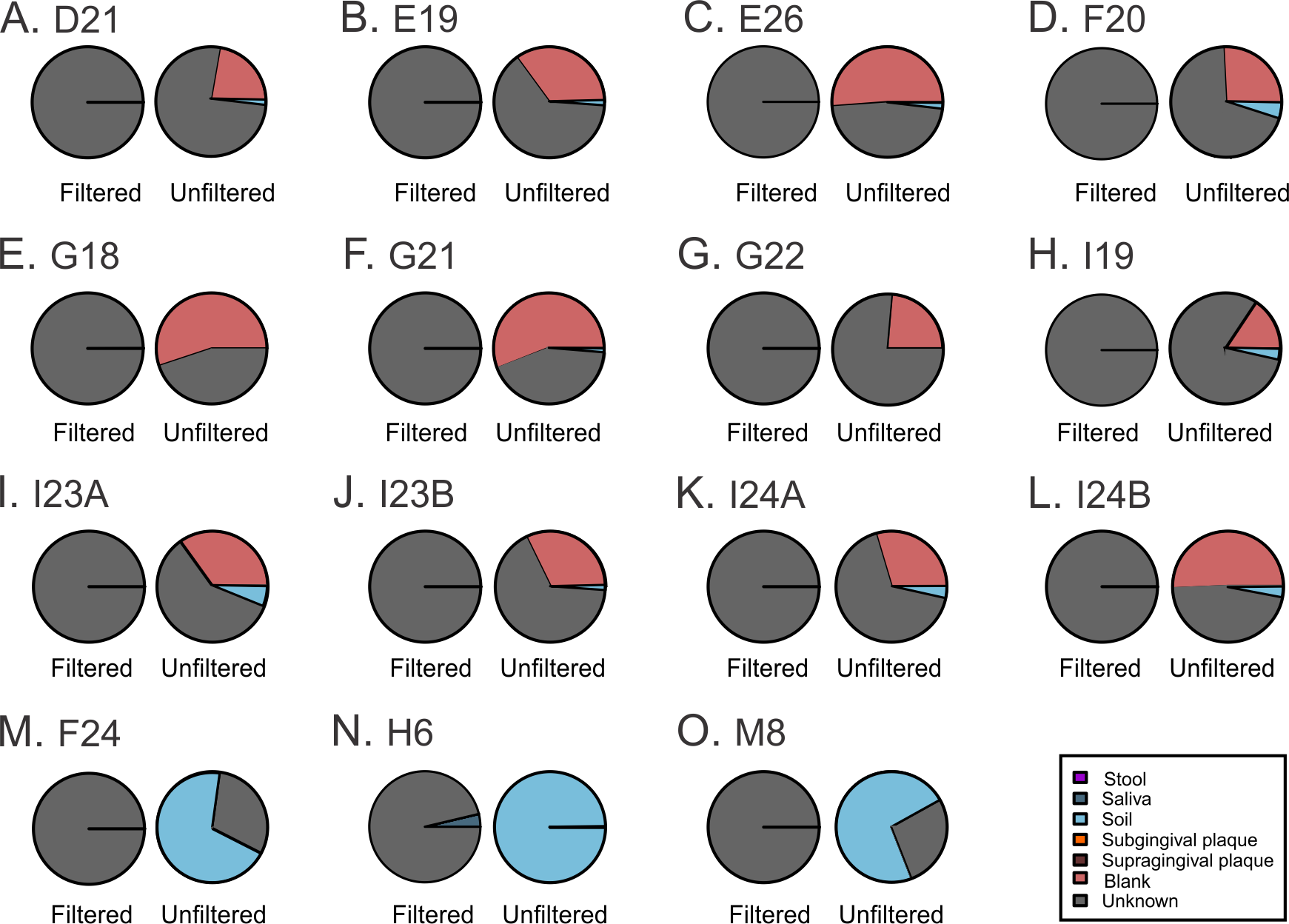

Supplement: Figure S1 — SourceTracker analyses of the dental calculi samples including D21 (A), E19 (B), E26 (C), F20 (D), G18 (E), G21 (F), G22 (G), I19 (H), I23A (I), I23B (J), I24A (K), I24B (L), F24 (M), H6 (N) and M8 (O). Dental calculi microbiomes were compared to stool, coprolite, saliva, soil from the archaeological site of Sorcé, subgingival plaque, and supragingival plaque microbiomes. Dental calculi sequences not matching any of the microbiomes included were classified as unknown. Figure shows results prior and after filtering soil and blank control OTUs from the dental calculi. [file peerj-05-3277-s001.png]

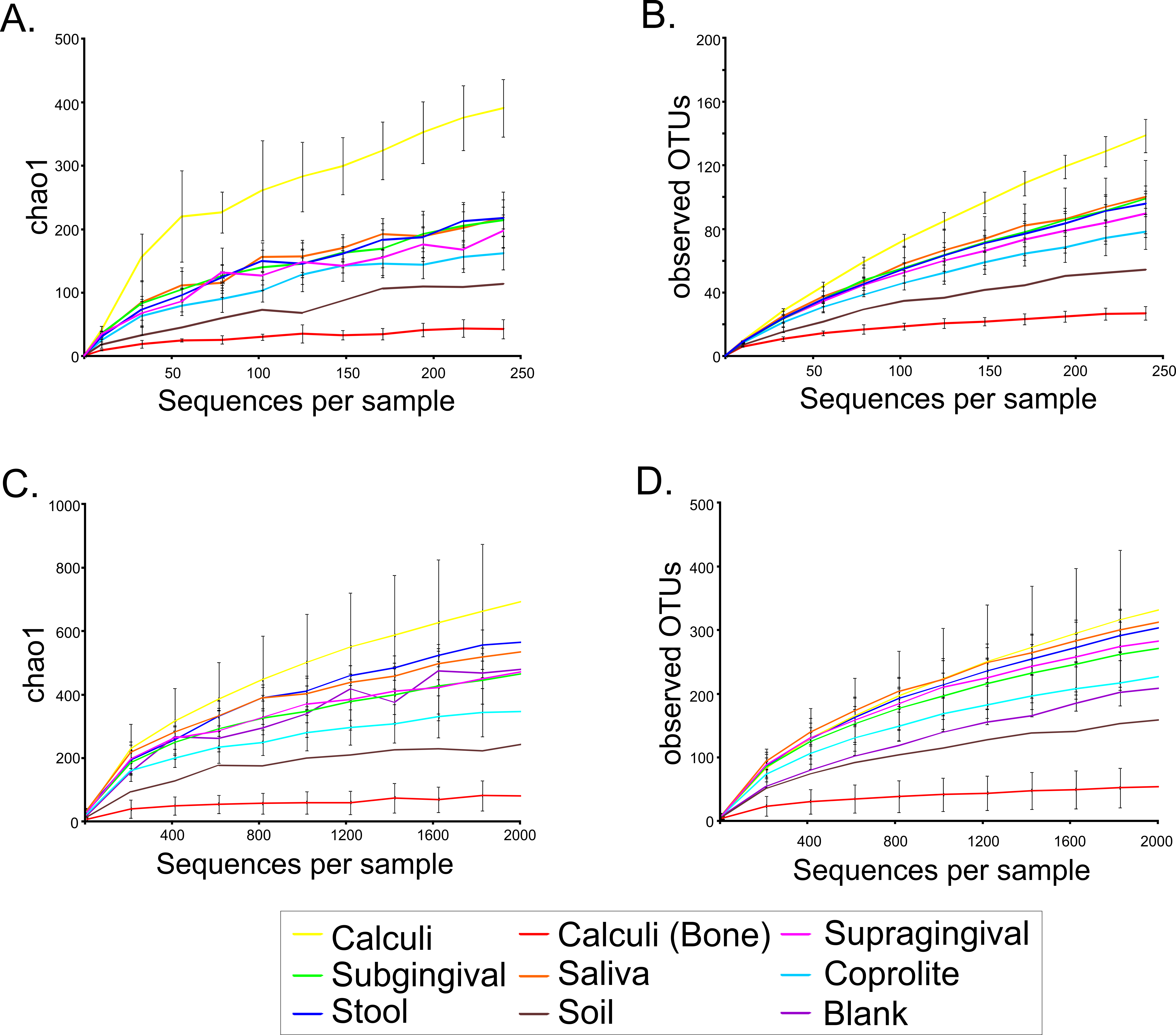

Supplement: Figure S2 — Alphararefaction curves of chao1 (A), and observed OTUs values (B) after filtering soil and blank control OTUs from the dental calculi, and chao1 (C), and observed OTUs (D) values prior filtering soil and blank control OTUs. Samples included dental calculi from loose teeth samples (yellow), dental calculi from teeth attached to bone (red), supragingival plaque (pink), subgingival plaque (green), saliva (orange), coprolites (light blue), stool (blue), soil from the archaeological site of Sorcé (brown), and blank control (purple). [file peerj-05-3277-s002.png]

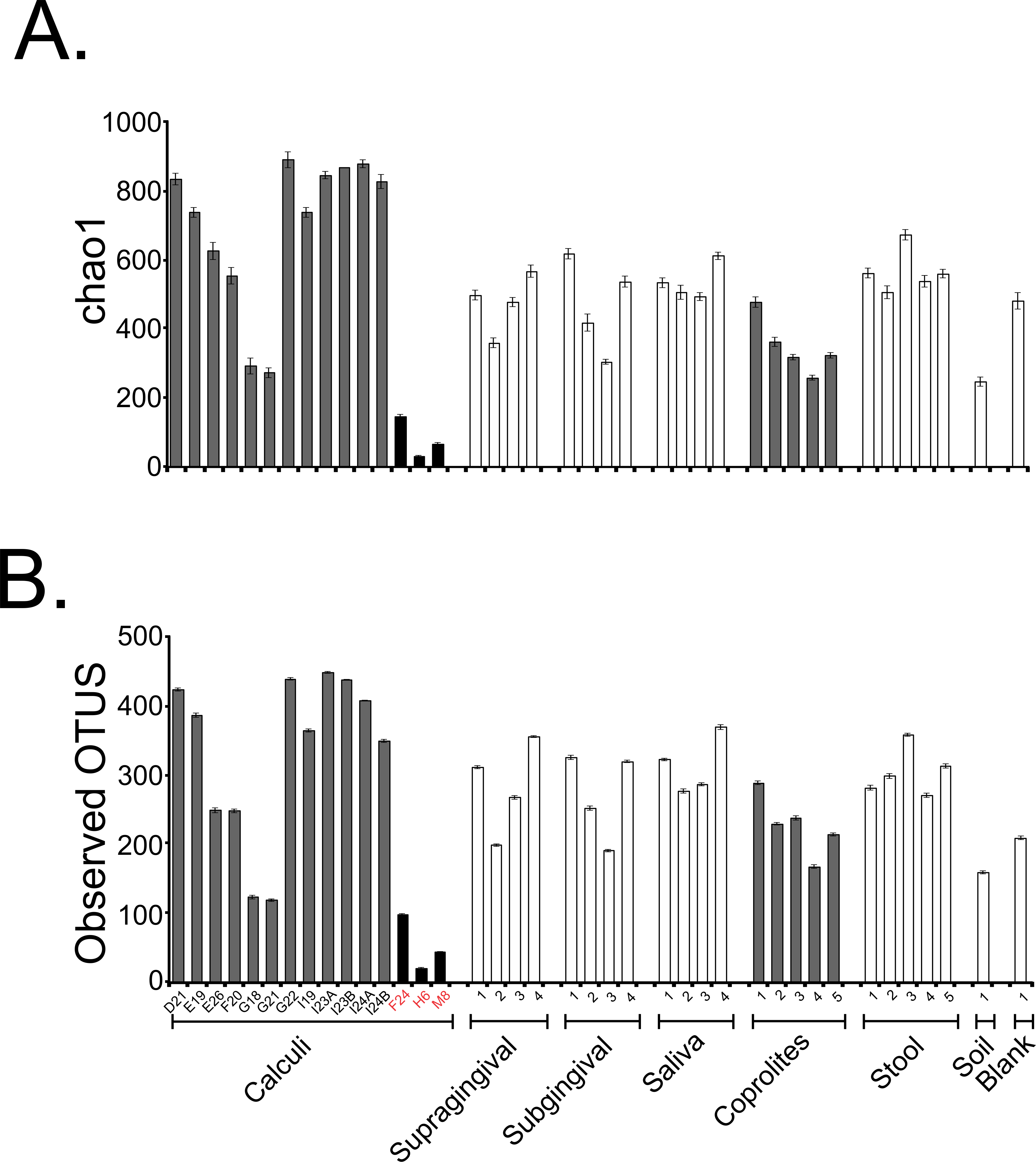

Supplement: Figure S3 — Bar plots of alpha diversity indices. Bar plots representing the chao 1 (A) and observed OTUs (B) indices for the bacterial taxonomy based on 16S rRNA gene of the dental calculi, modern supragingival and subgingival plaque, saliva, coprolites, stool and soil from the archaeological site of Sorcé microbiomes. Alpha diversity indices were computed from the average of ten iterations using the collate_α.py workflow. Soil and blank control OTUs were not filtered from the dental calculi prior analyses. [file peerj-05-3277-s003.png]

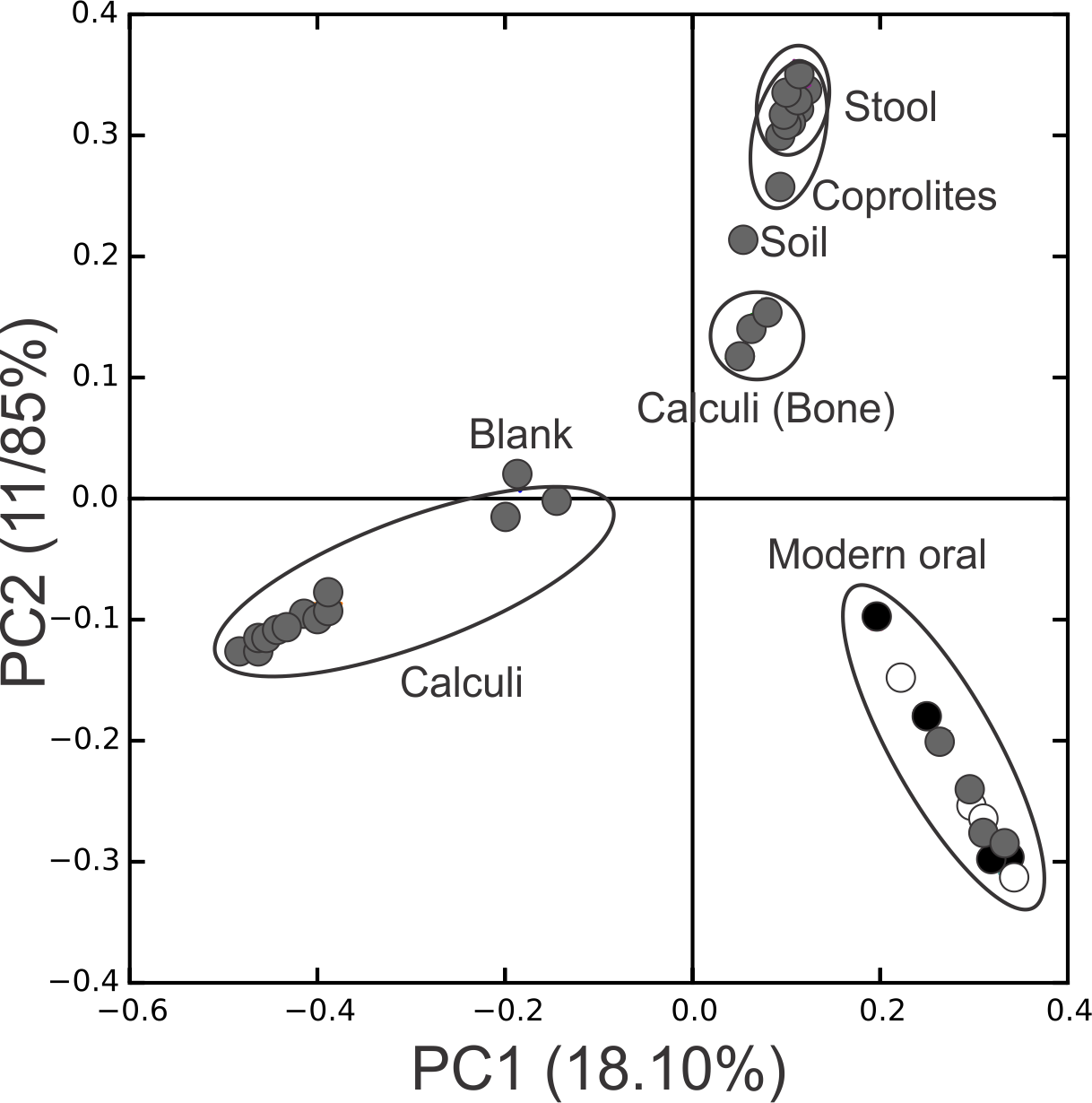

Supplement: Figure S4 — Principal Coordinates Analysis (PCoA) 2D plots of ancient and modern oral and gut microbiomes, as well as that of soil from the archaeological site of Sorcé. Dental calculi (yellow), dental calculi of teeth attached to bones that enabled the identification of gender or age (Dental calculi (Bone)) (red), coprolites (light blue), stool (dark blue), soil from the archaeological site of Sorcé (brown), supragingival (pink) and subgingival plaque (green), and saliva (orange). Soil and blank control OTUs were not filtered from the dental calculi prior analyses. [file peerj-05-3277-s004.png]

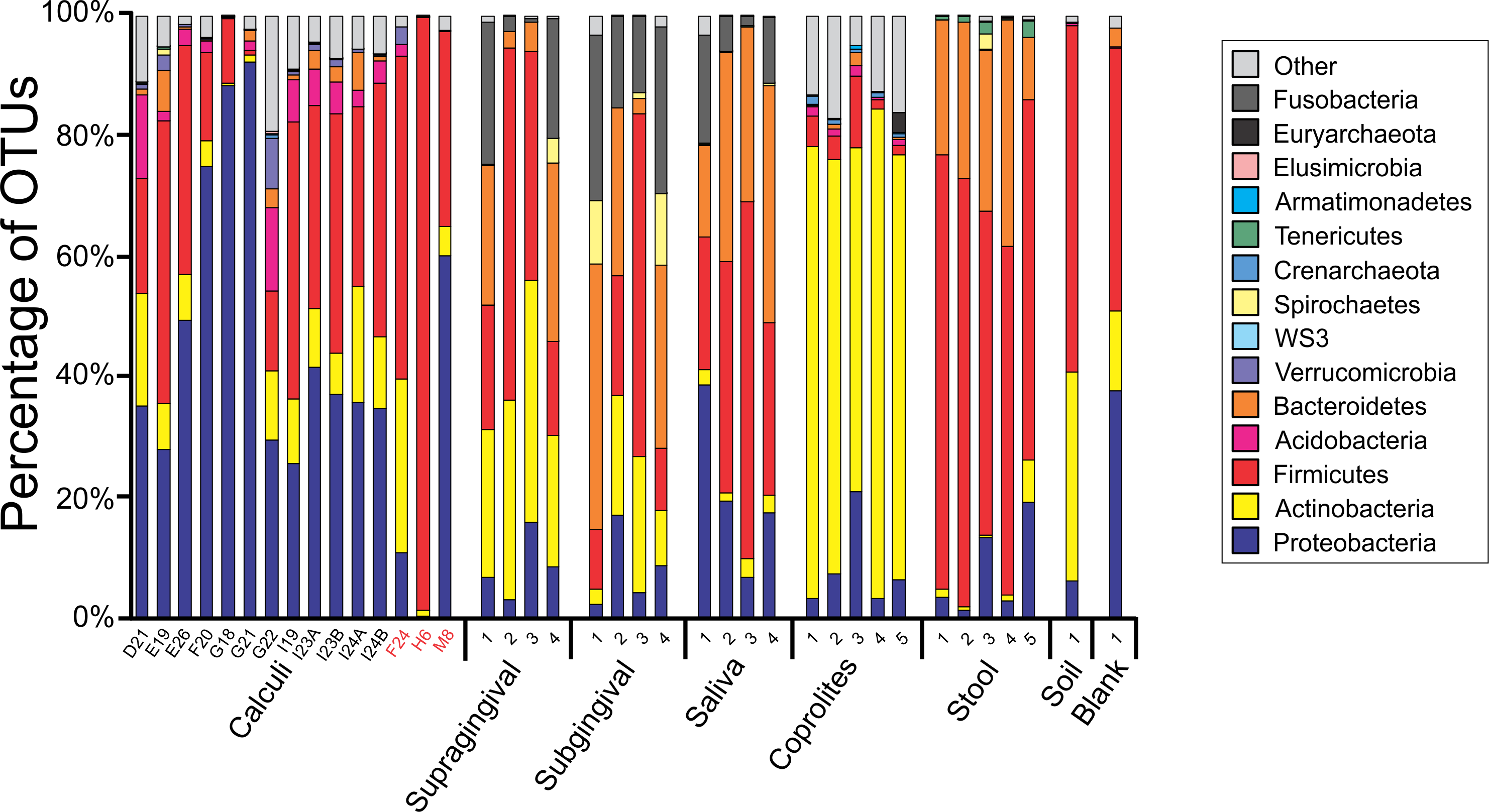

Supplement: Figure S5 — Barplots representing the bacterial taxonomy based on 16S rRNA gene. Data are shown at the phylum level for dental calculi, supragingival plaque, subgingival plaque, saliva, coprolites, stool, soil from the archeological site of Sorcé, and a blank control. Soil and blank control OTUs were not filtered from the dental calculi prior analyses. [file peerj-05-3277-s005.png]

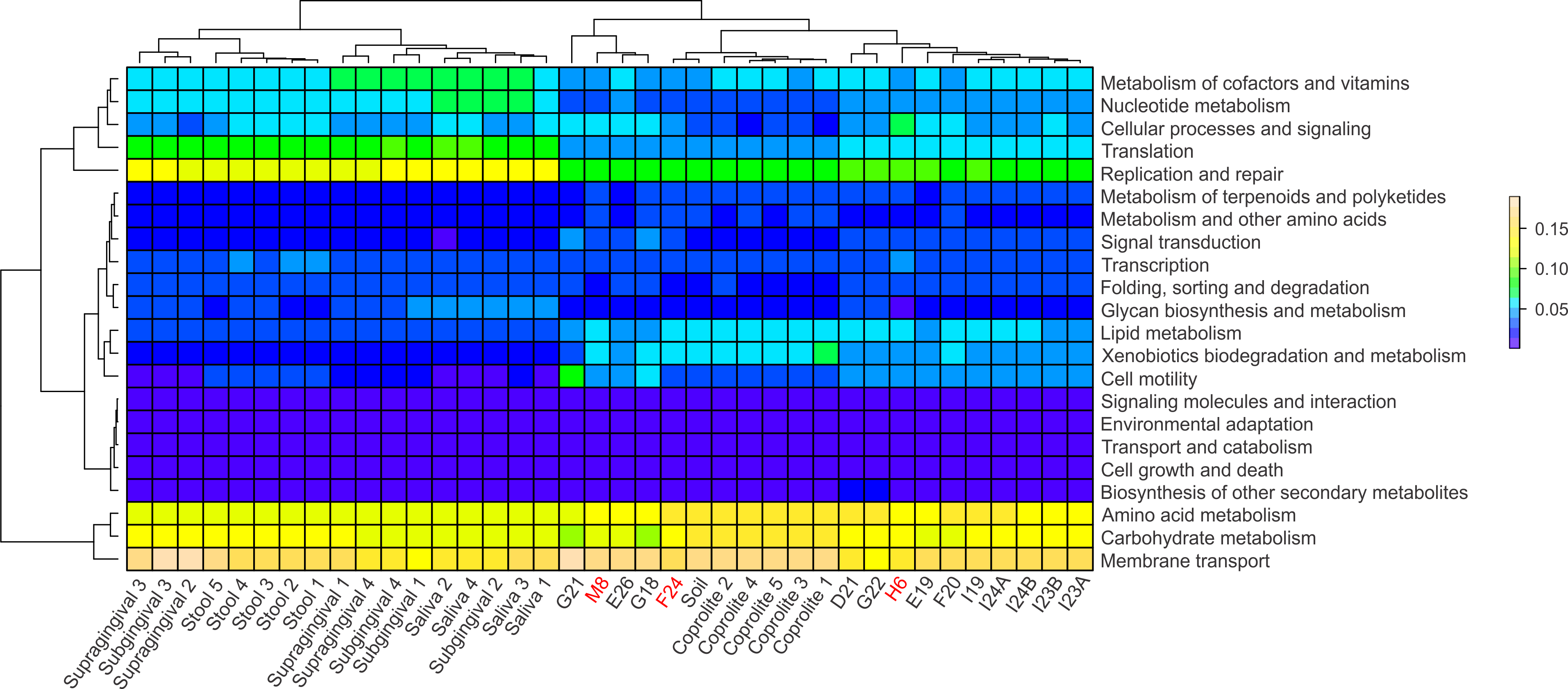

Supplement: Figure S6 — Heatmap of the relative abundances of the predicted functional categories (level 2) of microbiomes of dental calculi, coprolites, supragingival and subgingival plaque, saliva, stool and soil from the archeological site of Sorcé. Functional categories were predicted using PICRUSt. Soil and blank control OTUs were not filtered from the dental calculi prior analyses. [file peerj-05-3277-s006.png]

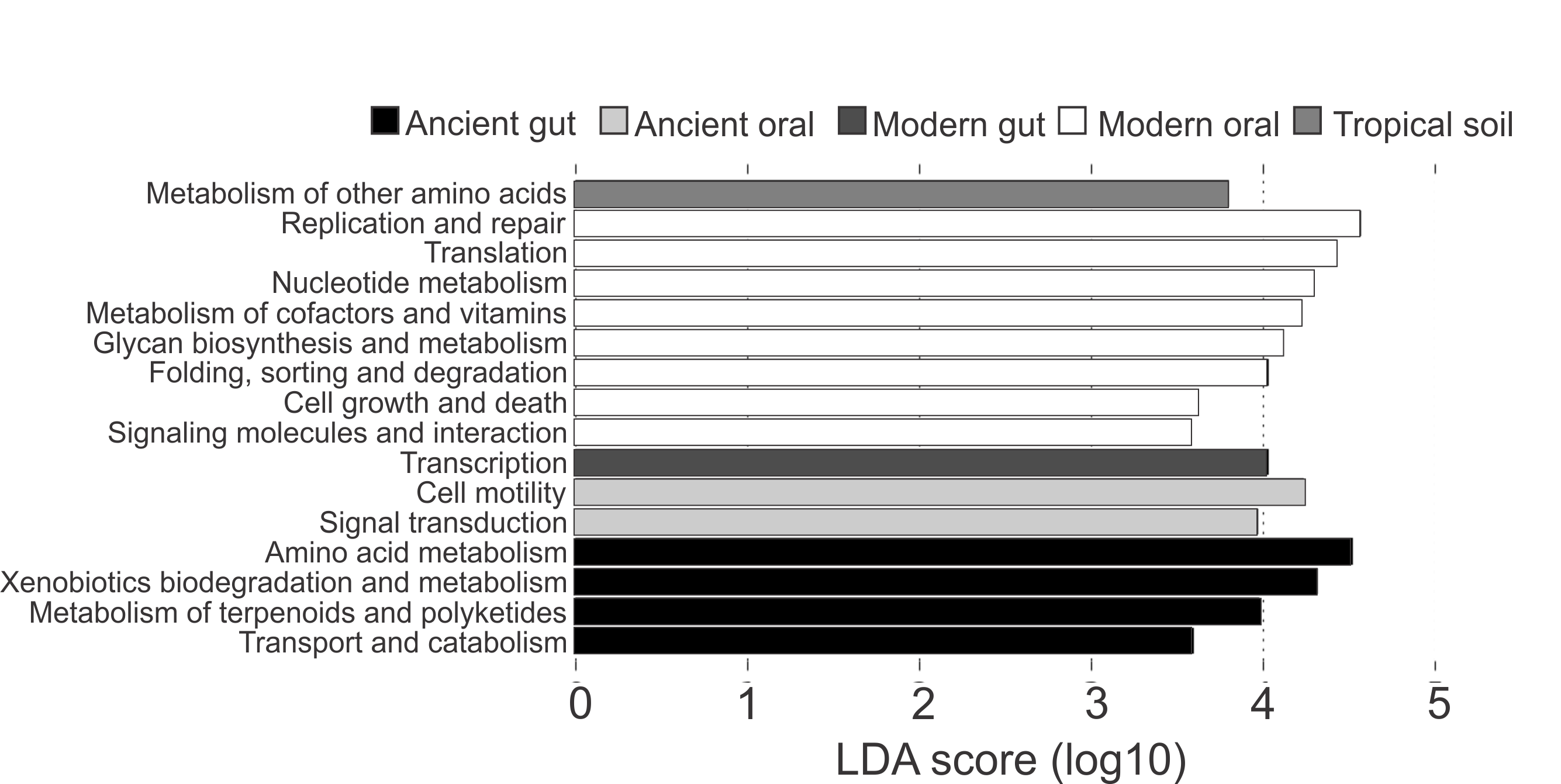

Supplement: Figure S7 — Linear discriminatory analyses. Effect size (LEfSe) plots of predicted functional categories (level 2). Functional categories of dental calculi (ancient oral microbiomes, green), supragingival plaque, subgingival plaque and saliva (modern oral microbiomes, purple), coprolites (ancient gut microbiomes, red), stool (modern gut microbiomes, blue) and soil from the archeological site of Sorcé (tropical soil, light blue) were predicted using PICRUSt. Soil and blank control OTUs were not filtered from the dental calculi prior analyses. [file peerj-05-3277-s007.png]
