# Supplementary material for: Insights of the dental calculi microbiome of pre-Columbian inhabitants from Puerto Rico
Source: PeerJ. 2017 May 2;5:e3277. doi: 10.7717/peerj.3277 (PMC5417066; doi:10.7717/peerj.3277)
Supplement: Table S1 [file peerj-05-3277-s017.doc]

**Table S1.** Sequence information. Modern sample sequences were downloaded from the Human Microbiome Project (HMP) or from NCBI. Values represent the number of sequences prior data rarefication.

| **Sample** | **Sequence read archive** | **Details** | **Number of sequences*** | **Number of OTUs** |
| --- | --- | --- | --- | --- |
| Supragingival1 | SRS013252 | Male | 5,704 | 460 |
| Supragingival2 | SRS042272 | Female | 6,389 | 331 |
| Supragingival3 | SRS020893 | Male | 4,147 | 336 |
| Supragingival4 | SRS022315 | Female | 4,681 | 492 |
| Subgingival1 | SRS021816 | Female | 3,695 | 416 |
| Subgingival2 | SRS022119 | Female | 4,364 | 355 |
| Subgingival3 | SRS014323 | Female | 3,863 | 259 |
| Subgingival4 | SRS023328 | Female | 4,455 | 428 |
| Saliva1 | SRS064596 | Male | 7,686 | 536 |
| Saliva2 | SRS017345 | Male | 4,934 | 391 |
| Saliva3 | SRS013228 | Male | 5,432 | 427 |
| Saliva4 | SRS013185 | Male | 4,690 | 507 |
| Stool1 | ERX115092 | Amazonian | 2,491,910 | 4047 |
| Stool2 | ERX115316 | Amazonian | 2,660,110 | 4506 |
| Stool3 | ERX115218 | Amazonian | 1,846,152 | 4263 |
| Stool4 | ERX115130 | Amazonian | 3,351,340 | 4883 |
| Stool5 | ERX115095 | Amazonian | 2,851,138 | 4375 |
| Coprolite1** |  | Saladoid | 43,856 | 975 |
| Coprolite2** |  | Saladoid | 57,408 | 936 |
| Coprolite3** |  | Saladoid | 42,346 | 668 |
| Coprolite4** |  | Saladoid | 3,298 | 318 |
| Coprolite5** |  | Saladoid | 93,594 | 745 |

*Number of sequences prior rarefaction.

** Data were obtained from the MG-RAST project 16S_Coprolite_Study.

Dental calculi samples highlighted in bold were attached to bone fragments that enabled the gender or approximate age determination.
