# Supplementary material for: Insights of the dental calculi microbiome of pre-Columbian inhabitants from Puerto Rico
Source: PeerJ. 2017 May 2;5:e3277. doi: 10.7717/peerj.3277 (PMC5417066; doi:10.7717/peerj.3277)
Supplement: Table S5 [file peerj-05-3277-s021.docx]

| **Group1** | **Group2** | **Group1 mean** | **Group1 std** | **Group2 mean** | **Group2 std** | **t stat** | **p-value** |
| --- | --- | --- | --- | --- | --- | --- | --- |
| Soil | Coprolite | 159.300 | 0.000 | 228.000 | 39.050 | -1.436 | 1.000 |
| Control | Coprolite | 209.400 | 0.000 | 228.000 | 39.050 | -0.389 | 1.000 |
| Subgingival plaque | Stool | 272.375 | 55.110 | 304.900 | 30.580 | -0.989 | 1.000 |
| Control | Calculi | 209.400 | 0.000 | 333.817 | 114.729 | -0.998 | 1.000 |
| Saliva | Calculi | 314.350 | 36.426 | 333.817 | 114.729 | -0.312 | 1.000 |
| Saliva | Stool | 314.350 | 36.426 | 304.900 | 30.580 | 0.373 | 1.000 |
| Control | Stool | 209.400 | 0.000 | 304.900 | 30.580 | -2.550 | 0.700 |
| Soil | Saliva | 159.300 | 0.000 | 314.350 | 36.426 | -3.297 | 1.000 |
| Saliva | Subgingival plaque | 314.350 | 36.426 | 272.375 | 55.110 | 1.101 | 1.000 |
| Control | Subgingival plaque | 209.400 | 0.000 | 272.375 | 55.110 | -0.885 | 1.000 |
| Saliva | Coprolite | 314.350 | 36.426 | 228.000 | 39.050 | 2.995 | 1.000 |
| Coprolite | Supragingival plaque | 228.000 | 39.050 | 283.900 | 58.047 | -1.518 | 1.000 |
| Saliva | Supragingival plaque | 314.350 | 36.426 | 283.900 | 58.047 | 0.770 | 1.000 |
| Coprolite | Subgingival plaque | 228.000 | 39.050 | 272.375 | 55.110 | -1.25 | 1.000 |
| Calculi | Supragingival plaque | 333.817 | 114.729 | 283.900 | 58.047 | 0.781 | 1.000 |
| Control | Soil | 209.400 | 0.000 | 159.300 | 0.000 | None | None |
| Calculi | Subgingival plaque | 333.817 | 114.729 | 272.375 | 55.110 | 0.965 | 1.000 |
| Coprolite | Calculi (Bone) | 228.000 | 39.050 | 54.567 | 32.439 | 5.602 | 0.630 |
| Saliva | Calculi (Bone) | 314.350 | 36.426 | 54.567 | 32.439 | 8.267 | 1.000 |
| Control | Calculi (Bone) | 209.400 | 0.000 | 54.567 | 32.439 | 3.375 | 1.000 |
| Control | Supragingival plaque | 209.400 | 0.000 | 283.900 | 58.047 | -0.994 | 1.000 |
| Coprolite | Calculi | 228.000 | 39.050 | 333.817 | 114.730 | -1.892 | 1.000 |
| Soil | Calculi (Bone) | 159.300 | 0.000 | 54.567 | 32.439 | 2.283 | 1.000 |
| Supragingival plaque | Stool | 283.900 | 58.047 | 304.900 | 30.580 | -0.615 | 1.000 |
| Soil | Supragingival plaque | 159.300 | 0.000 | 283.900 | 58.047 | -1.663 | 1.000 |
| Subgingival plaque | Supragingival plaque | 272.375 | 55.110 | 283.900 | 58.047 | -0.249 | 1.000 |
| Calculi (Bone) | Supragingival plaque | 54.567 | 32.439 | 283.900 | 58.047 | -5.206 | 1.000 |
| **Calculi** | **Calculi (Bone)** | **333.817** | **114.729** | **54.567** | **32.439** | **3.886** | **0.035** |
| Subgingival plaque | Calculi (Bone) | 272.375 | 55.110 | 54.567 | 32.439 | 5.154 | 1.000 |
| Soil | Subgingival plaque | 159.300 | 0.000 | 272.375 | 55.110 | -1.589 | 1.000 |
| Control | Saliva | 209.400 | 0.000 | 314.350 | 36.426 | -2.232 | 1.000 |
| Soil | Stool | 159.300 | 0.000 | 304.900 | 30.580 | -3.888 | 0.770 |
| Calculi | Stool | 333.817 | 114.729 | 304.900 | 30.580 | 0.522 | 1.000 |
| Calculi (Bone) | Stool | 54.567 | 32.439 | 304.900 | 30.580 | -9.487 | 0.105 |
| Soil | Calculi | 159.300 | 0.000 | 333.817 | 114.729 | -1.399 | 1.000 |
| Coprolite | Stool | 228.000 | 39.050 | 304.900 | 30.580 | -3.101 | 0.420 |

**Table S5:** Observed OTUs statistics. Statistically significant values are highlighted in bold. Dental calculi samples recovered from teeth attached to bone fragments that enabled gender or age determination are shown as Calculi (Bone). Results are representative of unfiltered OTUs from soil and blank controls.
